# Maternal diet modulates placental nutrient transporter gene expression in a mouse model of diabetic pregnancy

**DOI:** 10.1371/journal.pone.0224754

**Published:** 2019-11-27

**Authors:** Claudia Kappen, Claudia Kruger, Sydney Jones, Nils J. Herion, J. Michael Salbaum

**Affiliations:** 1 Department of Developmental Biology, Pennington Biomedical Research Center, Louisiana State University System, Baton Rouge, Louisiana, United States of America; 2 Baton Rouge, Louisiana, United States of America Regulation of Gene Expression Laboratory, Pennington Biomedical Research Center, Louisiana State University System, Baton Rouge, Louisiana, United States of America; Virgen Macarena University Hospital, School of Medicine, University of Seville, SPAIN

## Abstract

Diabetes in the mother during pregnancy is a risk factor for birth defects and perinatal complications and can affect long-term health of the offspring through developmental programming of susceptibility to metabolic disease. We previously showed that Streptozotocin-induced maternal diabetes in mice is associated with altered cell differentiation and with smaller size of the placenta. Placental size and fetal size were affected by maternal diet in this model, and maternal diet also modulated the risk for neural tube defects. In the present study, we sought to determine the extent to which these effects might be mediated through altered expression of nutrient transporters, specifically glucose and fatty acid transporters in the placenta. Our results demonstrate that expression of several transporters is modulated by both maternal diet and maternal diabetes. Diet was revealed as the more prominent determinant of nutrient transporter expression levels, even in pregnancies with uncontrolled diabetes, consistent with the role of diet in placental and fetal growth. Notably, the largest changes in nutrient transporter expression levels were detected around midgestation time points when the placenta is being formed. These findings place the critical time period for susceptibility to diet exposures earlier than previously appreciated, implying that mechanisms underlying developmental programming can act on placenta formation.

## Introduction

Exposure to maternal diabetes during pregnancy is linked to a higher risk for adverse health outcomes later in life [1–4]. It has been proposed that in such pregnancies, placental function may be compromised, possibly leading to altered placental hormone signaling [5, 6] and altered nutrient supply to the fetus [7, 8]. Employing a mouse model of diabetes, induced by Streptozotocin, we have previously shown that maternal diabetes attenuates weight gain during pregnancy [9], and that the disease was associated with reduced fetal growth [10]. This growth reduction was exaggerated by feeding to the dam a commercial diet specifically formulated to promote pregnancy and lactation in normal conditions. However, this diet produced worse outcomes than regular mouse chow in the context of maternal diabetes [10], including a higher rate of neural tube defects.

In diabetic females fed this diet, placenta weight during the second half of the pregnancy was also reduced, by as much as 15% [9]. It is plausible that impaired placental growth and function could be responsible for the observed fetal growth reduction. Under diabetic conditions, we found the spongiotrophoblast cell layer reduced in the placenta [11], associated with ectopic appearance of spongiotrophoblast clusters within the labyrinth, instead of their normal location in the junctional zone. Prior to overt histological changes, we detected altered gene expression in the diabetic placenta by midgestation [9]. The effects of diet and diabetes at the molecular level were complex, in that subsets of genes responded only to diet or to maternal diabetes, respectively, and other gene subsets to both conditions, in coordinated or opposing directions [9]. For example, in placentae of diabetic pregnancies, we detected increased expression of Prolactin 5a [11], which is associated with decreased trophoblast invasion and shallower placentation in rats [12]. Furthermore, we found reduced placental expression of the gene encoding pregnancy-associate plasma protein Pappa 2 [11], which was subsequently identified as a causative gene for short stature in humans [13], and for smaller body size in mice [14, 15]. Thus, our earlier gene expression assays provide evidence to implicate specific molecular pathways in the placental and fetal phenotypes observed in our mouse model in response to maternal diabetes.

To better understand the role of maternal diet in diabetic pregnancies, the present study examined the expression of nutrient transporters in our diabetic pregnancy model. Because amino acids transporters have been investigated previously in human and rodent placentae in the context of diabetes [16–19], we here focused our attention on transporters for glucose and fatty acids. Altered expression of nutrient transporters could provide a plausible explanation for the effects of maternal hyperglycemia and hyperlipidemia on placental and fetal growth in diabetic pregnancies, and for the aggravating effects of a diet with higher fat content.

## Materials and methods

The experimental model consisted of female mice of the FVB strain (Charles River). All experiments involving animals were conducted in accordance with the US National Academies' *Guide for the Care and Use of Laboratory Animals*, and in accordance and compliance with all applicable rules and regulations, and had been approved by the Institutional Animal Care and Use Committee at Pennington Biomedical Research Center.

The FVB females received the standard diet, Labdiet Purina 5001 (here termed chow) until they were divided into two experimental groups at 8 weeks of age: one group was continued on chow, while Purina 5015 (here termed breeder diet) was fed to the other group. In the Purina 5001 formulation, 28.5% of calories are derived from protein, 13.5% from fat, and 58% from carbohydrates (https://www.labdiet.com/cs/groups/lolweb/@labdiet/documents/web_content/mdrf/mdi4/~edisp/ducm04_028021.pdf), and Purina 5015 is composed of 19.8% of calories are derived from protein, 25.3% from fat, and 54.8% from carbohydrates (https://www.labdiet.com/cs/groups/lolweb/@labdiet/documents/web_content/mdrf/mdi4/~edisp/ducm04_028439.pdf). The details on diet formulations are available in the linked PDF documents from the manufacturer. Notable (more than 2-fold) differences between diets in micronutrients were in content of Fluorine (15 ppm vs. 6.5 ppm), Carotene (2.3 ppm vs. 0.2 ppm), and Folic Acid (7.1 ppm vs. 2.9 ppm). It is important to note that both diets are nutritionally replete for mice under normal conditions.

Diabetes was induced by Streptozotocin, which was injected i.p. at 100mg/kg body weight twice within a week. As previously described [9–11, 20], females whose blood glucose levels exceeded 250mg/dl were considered diabetic, and were set up for mating no earlier than 7 days after the last injection. The day of detection of a vaginal plug was counted as day 0.5 of gestation. This model has been described in detail [9], and the studies reported here used archived samples from the experiments described in that earlier report.

Tissue was removed at designated time points, and concepti were dissected as follows: Implantation sites were recovered from uteri, and embryos were removed, as well as surrounding membranes. The labyrinth and maternal tissue directly above it were taken as placenta samples. To achieve maximum consistency of tissue harvest over all time conditions and time points, all dissections were performed by the same experimenter.

Measurements of gene expression levels were performed by quantitative real-time PCR (Q-RT-PCR) as described in detail elsewhere [9, 21]. Table 1 lists the Primer pairs used in the present study. For size determination of amplicons, 1μl of amplification reaction sample was applied to a DNA 1000-chip for measurement in the Agilent 2100 Bioanalyzer. For each metabolic condition and diet modality, at least 6 samples were assayed. All PCR measurements were conducted on three aliquots of the same sample to provide technical replicates, and averages from the triplicates were normalized to the reference gene Polymerase Epsilon 4 (Polε4). This reference gene was used in our earlier work on placenta gene expression [9, 20] and does not change in expression level over the various conditions or time: Ct value averages for control samples (n = 6) were 24.23±0.52 (±standard deviation), 24.38±0.33, and 24.37±0.41 for control samples from females fed chow diet; and 24.22±0.55, 24.37±0.41, 24.21±0.38 for samples from diabetic females fed the chow diet. For samples from females fed Breeder diet, the cycle threshold averages for n = 6 on each plate were 24.38±0.65, 24.34±0.47, 24.32±0.48 for normal pregnancies, and 24.39±0.56, 24.36±0.57, 24.38±0.61 for diabetic females. Analysis by timepoint, regardless of diet or metabolic condition, yielded no variance across any of the reference gene samples. The approaches for analysis of the PCR results were described previously [9, 20], including calculation of amplification efficiencies, normalization to Polymerase Epsilon 4 (Polε4) expression, and fold-changes. Statistical tests (T-test and ANOVA with Bonferroni post-hoc correction) were performed on ΔC_t_ values for a group size of 6 per modality. Variance is expressed as standard deviation from the average for each experimental group throughout the manuscript.

**Table 1:** Properties of primers used for quantitative Real-time PCR assays. Amplification efficiencies (AE) were calculated over all samples and conditions.

Gene	ENSEMBL	Forward		Reverse		Exon-exon	
symbol	Gene ID	primer	Position	primer	Position	boundary	AE
Cd36	ENSMUSG00000002944	GAACCTATTGAAGGCTTACATCCAA	1063–1087	TCCAGTTATGGGTTCCACATCTAA	1134–1111	yes	1.85
Cpt1a	ENSMUSG00000024900	CCAAACCCACCAGGCTACAG	1625–1644	AACTGGCACTGCTTAGGGATGT	1702–1681	yes	1.90
Cpt1b	ENSMUSG00000078937	CCCGAGCAGTGCCGGGAAGC	1652–1671	GAAATGAGCCAGCTGTAGGG	1822–1803	yes	1.81
Cpt1c	ENSMUSG00000007783	GACAACAAGGAGACAGACCAACAT	2116–2139	CCTTCAGTAGAGCCTGGTGCTT	2191–2170	yes	1.88
Cpt2	ENSMUSG00000028607	TGGGCCAGGGCTTTGAC	1787–1803	ATGTTGTGGTTTATCCGCTGGTA	1901–1879	yes	1.89
Slc27a1	ENSMUSG00000031808	AGATTGCCCACAGCGTTTTC	1629–1648	CATCACTAGCACGTCACCTGAGA	1693–1671	yes	1.90
Slc27a2	ENSMUSG00000027359	CGTCACGGTCATTCAGTACATTG	1663–1685	TTGGTTTCTGCGGTGTGTTG	1727–1708	yes	1.85
Slc27a3	ENSMUSG00000027932	CCCGACCTCGGTTTCTCA	1939–1956	CATCCTAACCTTCTGCTGTTTGAA	2012–1989	yes	1.88
Slc27a4	ENSMUSG00000059316	CTTTGTGTACCCTATCCGTTTGG	1566–1588	GGCCTGGCTGACCTGGTT	1669–1652	yes	1.90
Slc27a5	ENSMUSG00000030382	CCTGAGCAACCAGAAGACAAGA	1262–1283	CATTTGCCCGAAGTCCATTG	1328–1309	yes	1.90
Slc27a6	ENSMUSG00000024600	CGTGACTGTGTTTCAGTACATTGG	1119–1142	CCTTCTCTCTGAGGCTGTTTGC	1184–1163	yes	1.87
Slc2a1	ENSMUSG00000028645	GGGCATGTGCTTCCAGTATGT	1498–1518	ACGAGGAGCACCGTGAAGAT	1569–1550	yes	1.90
Slc2a2	ENSMUSG00000027690	CCGGGATGATTGGCATGT	1109–1126	GTCATGCTCACGTAACTCATCCA	1202–1180	yes	1.89
Slc2a3	ENSMUSG00000003153	TTGCTCCGTTTTCATGACGAT	1161–1181	GGGCCAGGTCCAATCTCAA	1277–1259	yes	1.89
Slc2a4	ENSMUSG00000018566	CCGGCAGCCTCTGATCAT	1020–1037	CGACTCGAAGATGCTGGTTGA	1116–1096	yes	1.90
Slc2a5	ENSMUSG00000028976	GGCGCTGCAGAACACCAT	1800–1817	AAGGCGTGTCCTATGACGTAGAC	1877–1855	yes	1.90
Slc2a6	ENSMUSG00000036067	CAGCCTTCGTCCTCACTAACTACTT	1394–1418	GGCAGATGGCCGAGAAGAA	1480–1462	yes	1.88
Slc2a7	ENSMUSG00000062064	AGCCCCCGCTACACTCTGA	700–718	GCAGCCTCCTTAGAGCTTGTCT	766–745	yes	1.90
Slc2a8	ENSMUSG00000026791	TCGGTCACTGTGGGCATAATC	955–975	CCCTGCTCTGTCCATGATGA	1023–1004	no	1.87
Slc2a9	ENSMUSG00000005107	GGCCCTGAACACTGAGAATGAC	2475–2496	TACATCCAGGACAGACATCATCAA	2563–2540	no	1.89
Slc2a10	ENSMUSG00000027661	AAGCAGGTTCCCTCTAAACTTTGG	1788–1811	CATCCAGGCGATGGTACTGA	1852–1833	yes	1.87
Slc2a12	ENSMUSG00000037490	CAAAGGCGAACTATGTGAAAAACA	1897–1920	TTACACTCTGGGAGCTGCTCTTG	2008–1986	yes	1.87
Slc2a13	ENSMUSG00000036298	GACTCCTTCGGATCAAAATACCA	1412–1434	TGTAGCAGAAACCACAGTCTGGAT	1497–1474	yes	1.83
Pole4	ENSMUSG00000030042	CGGGACAGGAAGCCATCTT	269–287	AGCAGTAGGCATCTTTTGCGATA	346–324	yes	1.90

Gene names follow current nomenclature in the Figures (Slc2a and Slc27a designations, respectively), but older nomenclature (Glut and Fatp) is also used throughout the text to facilitate reading.

## Results

Implantation sites were dissected from pregnant mouse dams at various time points of gestation, embryos/fetuses were removed, and placental tissue was processed for RNA preparation and RT-PCR assays. Expression was reliably detected for glucose transporter family members Slc2a1/Glut1, Slc2a3/Glut3, Slc2a5/Glut5, Slc2a6/Glut6, Slc2a8/Glut8, Slc2a9/Glut9, Slc2a10/Glut10, Slc2a12/Glut12, and Slc2a13/Glut13. Reactions specific for Slc2a2/Glut2, Slc2a4/Glut4, and Slc2a7/Glut7 did not yield amplicons of the sizes predicted for mRNA (as determined by Agilent chip); thus, Slc2a2/Glut2, Slc2a4/Glut4, and Slc2a7/Glut7 mRNA expression was undetectable in our placenta samples. All 6 fatty acid transporters assayed (Slc27a1 through Slc27a6, commonly known as Fatp1 through Fatp6) were detected, as were Carnitine palmitoyl transferases 1a, 1b, 1c and 2 (Cpt1a, Cpt1b, Cpt1c, Cpt2), as well as fatty acid transferase CD36.

### Nutrient transporter gene expression in placenta during murine pregnancy

**Fig 1:**
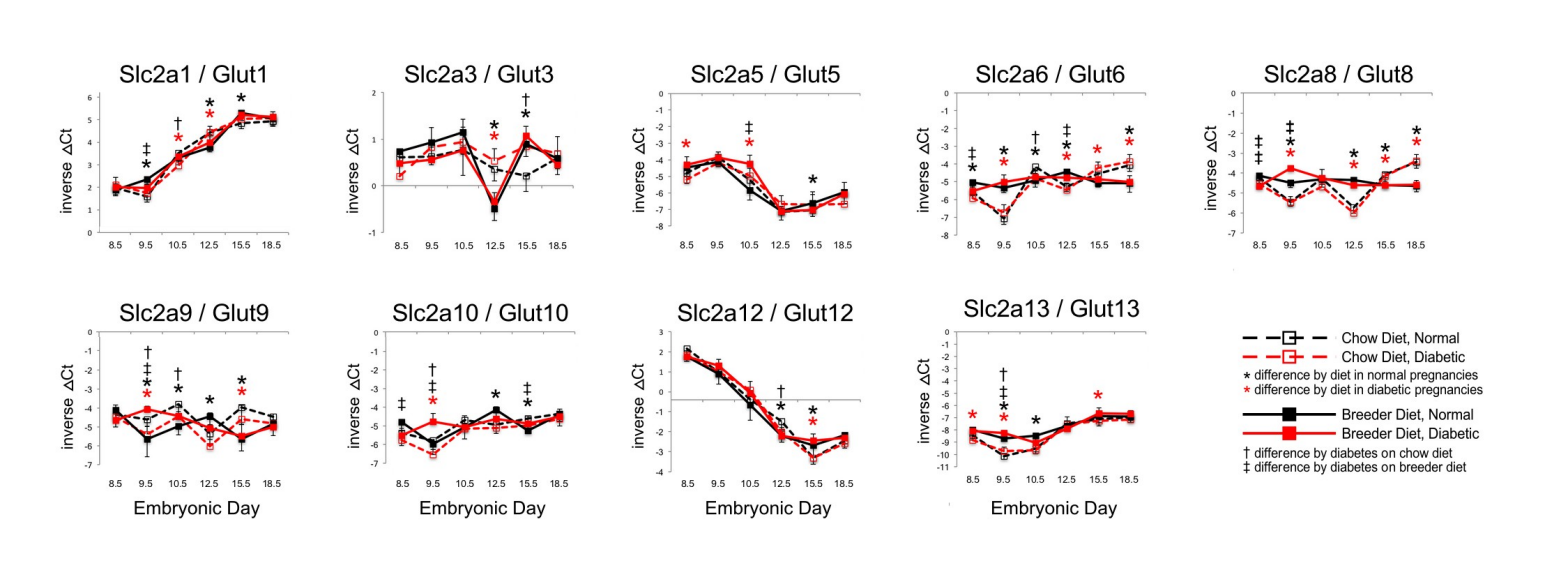
Expression of placental glucose transporters as influenced by maternal diabetes and diet. Expression levels are depicted as inverse ΔCt values to visually align the highest expression levels with the highest points on the graphs. Negative values indicate expression levels lower than the reference gene Polε4, positive values indicate higher expression relative to the reference. Each increment on the Y-axis depicts a two-fold difference (the equivalent of one PCR cycle). The sample number in each experimental group was n = 6. Metabolic state is depicted in black (normal) and red (diabetic), respectively, with significant difference between averages marked by daggers. Maternal diet is shown in solid symbols and lines (breeder diet), and open symbols with broken lines (chow diet), respectively, with significant differences marked by asterisks.

**Fig 2:**
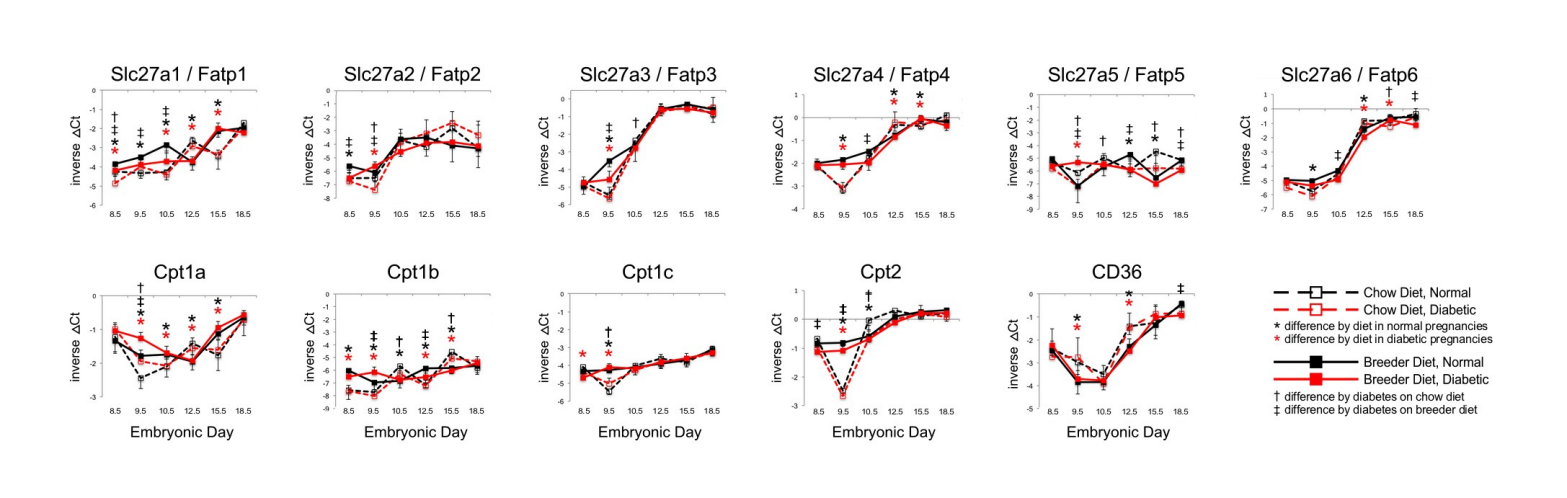
Expression of placental fatty acid transporters as influenced by maternal diabetes and diet. Data are depicted in the same manner as described in the Legend to Fig 1.

The temporal profiles for all genes that were detected in the RT-PCR assay are depicted in Figs 1 and 2 (Fig 1 for glucose transporters, Fig 2 for fatty acid transporters), with each increment on the Y-axis representing a two-fold difference (i.e. one PCR cycle). Slc2a5/Glut5 and Slc2a12/Glut12 had higher expression levels in placenta at early timepoints, which decreased towards the end of pregnancy by approximately 4-fold and 32-fold, respectively. Increasing expression over time was evident for many of the transporters, including Slc2a1/Glut1 (8-fold), Slc2a13/Glut13 (2-fold), CD36 (2-fold overall, 8-fold from day E9.5), Slc27a1/Fatp1 (4-fold), Slc27a2/Fatp2 (4-fold), Slc27a3/Fatp3 (more than 10-fold), Slc27a4/Fatp4 (4-fold), and Slc27a6/Fatp6 (more than 10-fold). Notably, Slc27a3/Fatp3 and Slc27a6/Fatp6 levels increased by more than 10-fold between E9.5 and E18.5, and Slc2a1/Glut1 expression increased by 8-fold. Moderate increases (approximately 2-fold) in expression were measured for the Cpt genes, of which Cpt1a and Cpt2 exhibited the highest overall expression levels. Thus, the genes encoding different transporter molecules responded in different manner over the time course of pregnancy, suggesting that they are regulated by different mechanisms, possibly by distinct transcription factors.

### Influence of maternal diabetes on placental nutrient transporter gene expression

Interestingly, only few nutrient transporter genes exhibited a specific response to the diabetic condition, and only at selected time points. Slc2a1/Glut1 expression levels were lower in diabetic placentae at E9.5, but only in dams fed the breeder diet; at E10.5, diabetic placentae had lower levels of Slc2a1/Glut1 only in dams fed the chow diet. Towards the end of pregnancy, Slc2a1/Glut1 levels were the same between all four experimental groups. Similar timepoint-specific responses were observed for Slc2a3/Glut3 at E15.5, Slc2a5/Glut5 at E10.5, Slc2a12/Glut12 at E12.5, and Slc2a13/Glut13 at E9.5. Slc2a6/Glut6, Slc2a8/Glut8, Slc2a9/Glut9 and Scl2a10/Glut10 all exhibited differences by metabolic state at some, but not all, timepoints. Overall, the differences ranged from two- to four-fold. Similar to Slc2a1/Glut1, the expression levels were normal at E18.5 for Slc2a3/Glut3, Slc2a5/Glut5, Slc2a9/Glut9, Slc2a10/Glut10, Slc2a12/Glut12 and Slc2a13/Glut13, while Slc2a6/Glut6 and Slc2a8/Glut8 displayed different expression levels by E18.5 that were solely driven by diet.

Of the fatty acid transporter genes (Fig 2), all responded to the metabolic condition at some time point, with Slc27a4/Fatp4 displaying lower expression at E10.5, Cpt1a and Cpt1c both exhibiting higher expression in diabetic placentae at E9.5 with chow diet, and Cpt1a also with breeder diet. The other fatty acid transporters showed variable response patterns to diabetes over time, mostly in dams fed breeder diet, but not necessarily in consistent fashion. For example, Slc27a5/Fatp5 had higher expression in diabetic dams on breeder diet at E9.5, and lower expression in diabetic dams fed chow, but at E18.5, expression was lower in diabetic placentae with both diets. CD36 and Slc27a6/Fatp6 also displayed lower expression in diabetic dams fed breeder diet at E18.5. Overall, differences in expression levels of fatty acid transporters, as influenced by diabetes, were no larger than two-fold.

### Influence of maternal diet on placental nutrient transporter gene expression

Alterations of expression levels by diet were observed for all Slc2as/Gluts; greater than two-fold were only observed for Slc2a3/Glut3 at E12.5, for Slc2a6/Glut6 at E9.5 and E18.5, for Slc2a8/Glut8 at E9.5, E12.5 and E18.5, and for Slc2a13/Glut13 at E9.5, regardless of diabetes exposure. However, the direction of change was not always consistent: for example, Slc2a3/Glut3 expression levels were higher in placentae from chow-fed dams than in dams on breeder diet at E12.5, whereas chow feeding resulted in lower levels for Slc2a6/Glut6 and Slc2a8/Glut8 at the same time point. Yet, at E18.5, Slc2a6/Glut6 and Slc2a8/Glut8 levels in chow-fed placentae were higher than in normal pregnancies. The diet effects at earlier time points were compensated for all other glucose transporter genes by E18.5, resulting in expression levels that were indistinguishable between experimental groups at that time point.

For the fatty acid transporter CD36, breeder diet lowered expression from E9.5 to E15.5. Patterns for other fatty acid transporters were variable, although at early timepoints, expression levels tended to be higher in placentae from breeder diet-fed dams (solid lines) for Slc27a1/Fatp1 through Slc27a6/Fatp6, as well as the mitochondrial acylcarnitine transporters Cpt1a, Cpt1b, Cpt1c and Cpt2. Except for the influence of diabetes on Cd36, Scl27a5/Fatp5 and Slc27a6/Fatp6 at E18.5, the effects of diet on fatty acid transporter expression levels were normalized towards the end of pregnancy. These results suggest the possibility that each gene could be uniquely responsive to the combination of diet and diabetes at different time points.

### Interaction of maternal diabetes and diet on nutrient transporter gene expression

**Fig 3:**
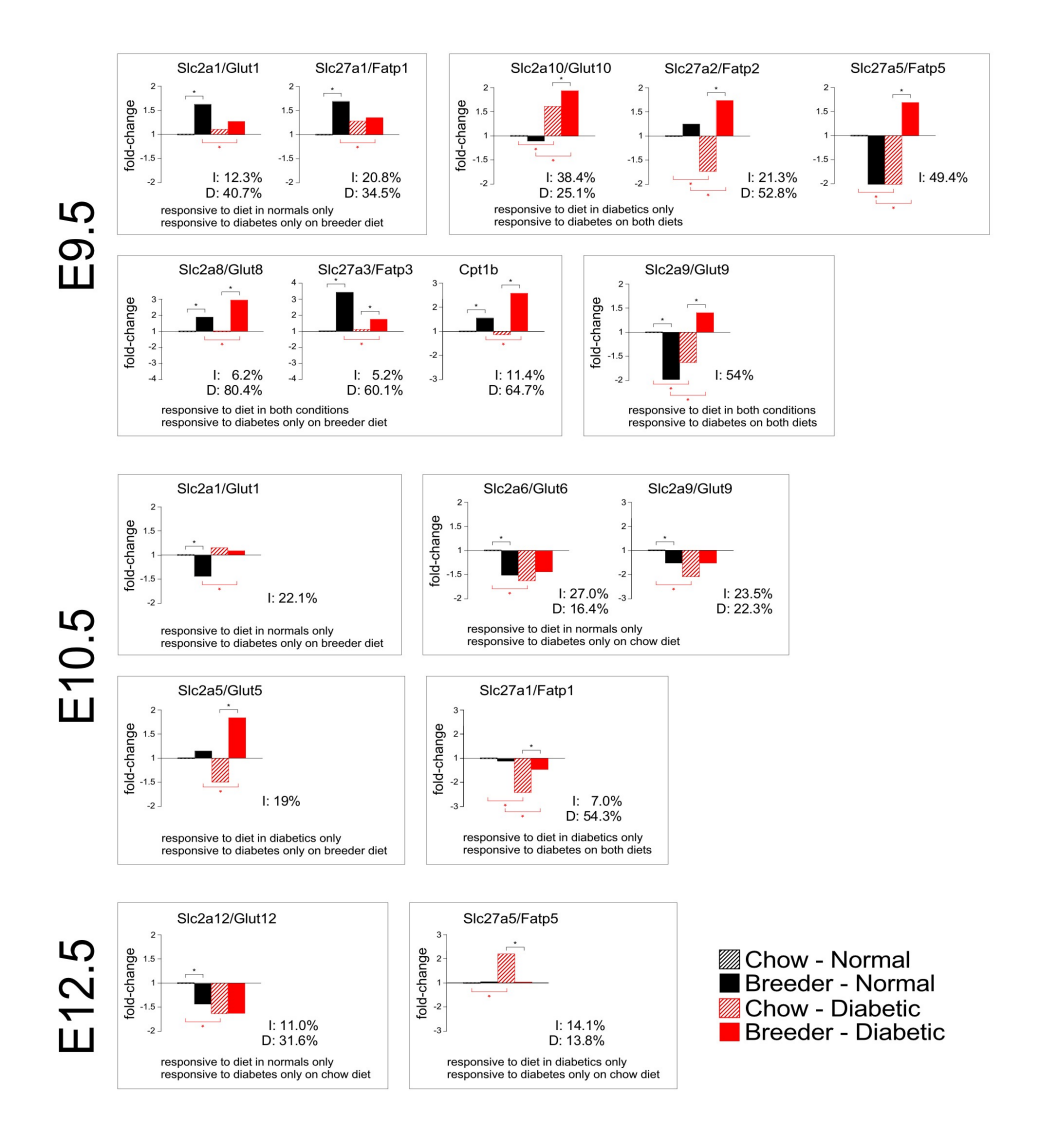
Interactions of maternal diet and diabetes on nutrient transporter gene expression in the placenta. Gene expression levels were determined by quantitative real-time PCR (data shown in Fig 1 and Fig 2). ‘‘Fold-change‘‘ of expression (expressed on the Y-axis) was calculated from the ΔCt-values relative to the expression levels in placentae from normal dams on chow diet. Note that the dimensions along the Y-axis may differ between panels. Asterisks indicate statistically significant differences between groups, as taken from Fig 1 and Fig 2. Two-factor ANOVA was performed using n = 6 samples for each condition, to detect statistically significant interactions between maternal diabetes and diet, and the variation explained by interactions -when found significant- is given in each respective graph. The extent of variation explained by maternal diet is also listed (D: Diet).

This prompted us to investigate the interaction of diet and metabolic status by 2-way ANOVA analysis. Fig 3 displays results for timepoint E9.5, selected from those results that exhibited significant interactions between diet and disease. Four patterns of responses were observed: genes that responded to diet or diabetes in only one condition (Slc2a1/Glut1 and Slc27a1/Fatp1); genes that responded to diet in one condition and to diabetes on both diets (Slc2a10/Glut10, Slc27a2/Fatp2 and Slc27a5/Fatp5); genes that responded to diet in both metabolic conditions but to diabetes only on breeder diet (Slc2a8/Glut8, Slc27a3/Fatp3 and Cpt1b); Slc2a9/Glut9 expression was significantly different between all 4 conditions. Notably, in the diabetic state, breeder diet always boosted gene expression, but there was no such consistent effect in normal pregnancies. Thus, for some genes, diet and diabetes exert concerted effects on expression, at least at E9.5 before the placenta becomes fully functional. However, it should be noted that the variation attributable to interaction varied considerably between genes. Also notable: when diabetes and diet were compared, diet was always the stronger and statistically significant contributor to the interaction.

**Fig 4:**
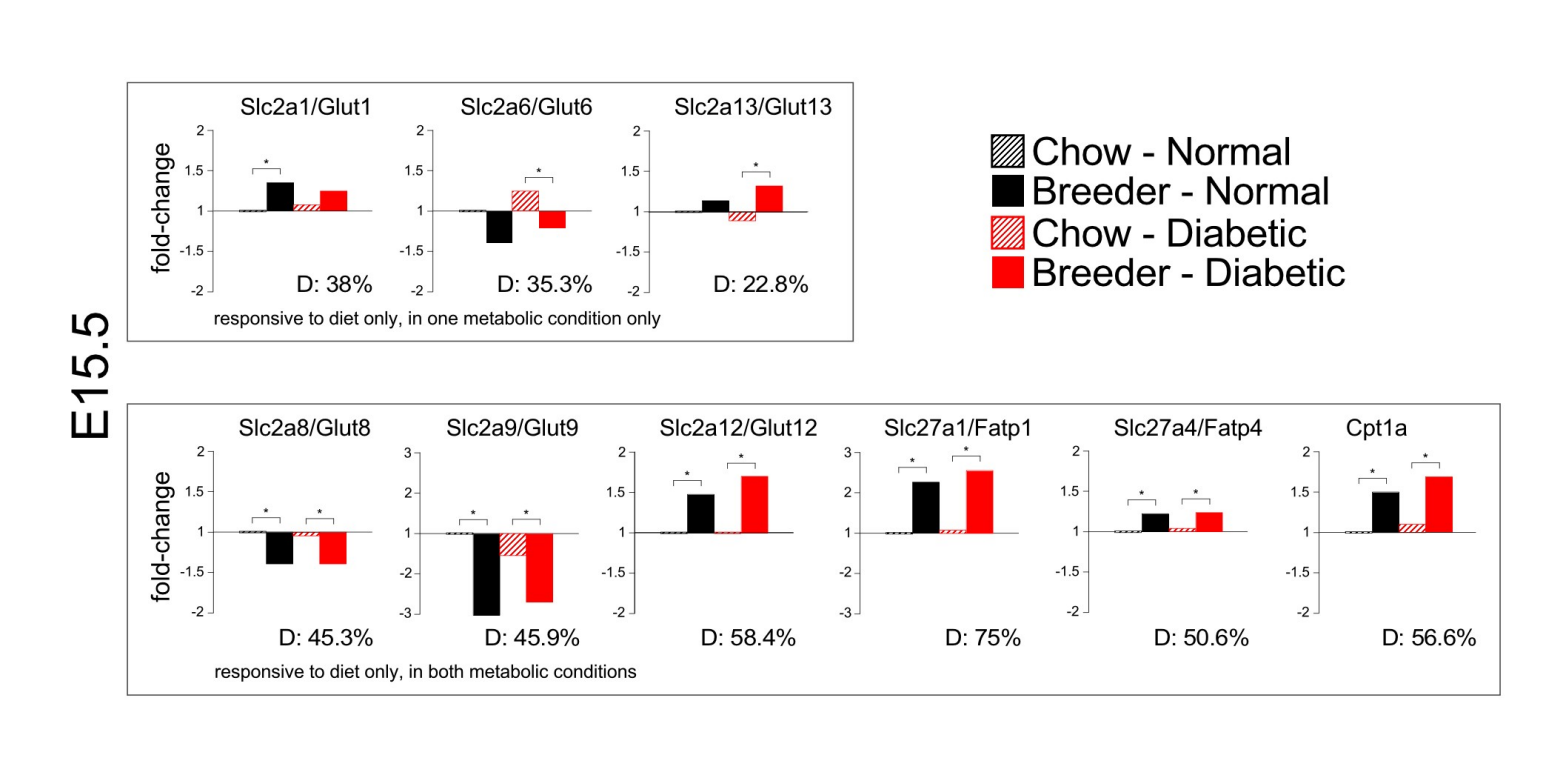
Effects of diet on nutrient transporter gene expression in the placenta. Data are depicted in the same manner as described in the Legend to Fig 3. The extent of variation explained by maternal diet is listed (D: Diet).

Interactions were also detected at E10.5, but by E12.5 only Slc2a12/Glut12 and Slc27a5/Fatp5 exhibited significant interactions. No significant interactions were detected by E15.5 or E18.5. The strong effect of diet was consolidated by E15.5 (Fig 4), when breeder diet caused elevated expression of Slc2a1/Glut1, Slc2a12/Glut12, Slc2a13/Glut13, Slc27a1/Fatp1, Slc27a4/Fatp4, and Cpt1a, and decreased the levels of Slc2a6/Glut6, Slc2a8/Glut8 and Slc2a9/Glut9.

Furthermore, despite absence of statistical interactions, effects of diabetes were significant at E9.5 for expression levels of Cpt1a (27.8%), Slc27a6/Fatp6 (10.8%), Slc2a13/Glut13 (7.2%), Cpt1c (6.4%), Slc2a6/Glut6 (3.2%) and Cpt2 (1.95%), but diet influence was much stronger for these genes with 48.5%, 51.4%, 83.8%, 80.4%, 84.2%, and 94.1% of variation attributable to diet, respectively). In addition, CD36 and Slc27a4/Fatp4 expression levels, while not influenced by diabetes, were strongly responsive to diet (40.6% and 82.1%, respectively). At E10.5, effects of diabetes were significant for Slc27a6/Fatp6, Slc27a4/Fatp4, and Cpt2 (27.8%, 19.4%, and 19.7%, respectively), and for Cpt1a, Slc2a13/Glut13, and Cpt1b, diet was a significant contributor (42.9%, 38.7%, and 31%, respectively).

Thus, while diabetes affects gene expression at early pregnancy time points, diet emerges as the strongest modifier of nutrient transporter expression in the placenta, even under conditions of uncontrolled maternal diabetes in our mouse model.

## Discussion

### Modulation of glucose transporter gene expression in mouse placenta by diabetes and diet

A major finding of this study is that glucose transporter expression levels in the placenta were more affected by diet than by diabetes in the pregnant mouse dam. This result is parallel to several studies in the rat that found no difference in placental Slc2a1/Glut1 expression in the diabetic animal [22–24]. In contrast, a study with small sample number (n = 3) reported reduced Slc2a1/Glut1 expression at the RNA and protein levels in diabetic placenta of ICR mice at E12 [25]; and in the non-obese diabetic (NOD) mouse strain, differences in RNA but not protein levels were detected [26]. Data for protein expression are currently not available from our experimental model. Contrasting findings in the literature could be related to mouse strain or timepoint of investigation, as well as mode and severity of diabetes. In human pregnancies with pre-gestational diabetes, SLC2A1/GLUT1 expression was found increased in term placenta [27], specifically in basal membrane preparations [28, 29]; however, the much longer pregnancy in human precludes a direct comparison to rodent pregnancies. Overall, our finding of increased placental Slc2a1/Glut1 expression over time in mouse placenta conforms to previous reports for human placental SLC2A1/GLUT1 expression [30], for sheep pregnancy [31, 32] and for rat [24] and mouse [33, 34].

Besides Slc2a1/Glut1, Slc2a3/Glut3 is the transporter gene with highest expression level in the placenta throughout pregnancy, but it was largely unaffected by maternal diabetes in our mice. This is in contrast to the rat model of STZ-induced diabetic pregnancy, where elevated Slc2a3/Glut3 expression at E20 was reported, both at the mRNA and protein levels [22]. In human pregnancies with pre-existing diabetes, reduction of SLC2A3/GLUT3 expression towards the term was seen [35]. Reduction of placental Slc2a3/Glut3 expression was also observed in caloric restriction paradigms in mouse [36] and rat [37]. Genetic reduction of Slc2a3/Glut3 expression by disruption of one allele in heterozygous mouse embryos was associated with reduced embryo growth after implantation [38], and homozygous deficiency of Slc2a3/Glut3 caused midgestation embryonic lethality, associated with ectodermal cell death [39], possibly as a result of insufficient glucose transport by the visceral endoderm [40]. We found Slc2a3/Glut3 expression reduced specifically in dams fed the breeder diet, but only on day E12.5. Reduction of overall Slc2a3/Glut3 levels at this timepoint was also reported for the ICR mouse strain, and was associated with a switch in mRNA splice forms from a longer 4.1kb to a shorter 2.7 kb size [33]. It is intriguing that breeder diet appears to have a stronger effect on this switch than chow diet, particularly in light of the finding that placental Slc2a3/Glut3 expression is sensitive to Igf2 levels [41].

We did not detect expression of Slc2a2/Glut2, nor of the Slc2a4/Glut4 gene. Both were initially thought absent from human placenta as well [42]. However, SLC2A4/GLUT4 expression was later reported in stromal cells in human placenta [43], in first trimester human syncytiotrophoblasts [44]; Slc2a4/Glut4 expression was also reported in rabbit [45], bovine [46], sheep [47], and rat placenta [48]. Mouse knockout experiments show that Slc2a4/Glut4 function is not required in the placenta for successful pregnancy [49].

Much less prior evidence is available for other members of the Slc2a gene family. Slc2a5/Glut5 was not detected in the rat uteroplacental unit before E8, and thereafter was present at low levels [50]. Slc2a5/Glut5 levels in our mouse model decreased during pregnancy, intermittently exhibiting a response to metabolic condition (at E12.5) and diet (at time points E8.5, E12.5, E15.5). As Slc2a5/Glut5 knockout mice are viable and fertile [51], the physiological role of Slc2a5/Glut5 in the placenta remains to be elucidated.

Slc2a6/Glut6 and Slc2a8/Glut8 were the only Slc2a family members in our study with changes in placental expression late in gestation, at E18.5. Both genes were expressed at relatively low levels, which might prompt questions about potential functional roles. On the other hand, given the cellular complexity of the placenta, with contributions of embryo-derived cells as well as maternal cells, particularly of hematopoietic origin, it is possible that low mRNA expression levels in whole tissue extracts reflect low abundance of a specific cell type that might be expressing a given nutrient transporter at appreciable levels, when considered on a per-cell basis. Slc2a8/Glut8 has been detected in sheep [52], with decreased expression in a placental insufficiency model, and increased expression in placentae of cortisol-infused ewes [53], a model of stress during pregnancy. In humans, IUGR pregnancies were associated with increased SLC2A8/GLUT8 expression in the maternal compartment of the placenta [54]. Analyses of Slc2a8/Glut8 knockout mice established a role in oocyte and blastocyst metabolism, in the decidualization reaction, and subsequent embryonic and postnatal growth [55], with Slc2a8/Glut8 deficiency resulting in smaller litters [56, 57]. Notably in our studies, placental Slc2a8/Glut8 levels were reduced in breeder diet-fed mice, which had smaller placentae regardless of maternal metabolic disease [9], and also produced smaller fetuses, exaggerated by maternal diabetes [10]. Apparently, the regulation of Slc2a12/Glut12 by Slc2a8/Glut8 as observed in enterocytes [58] is not reproduced in placental cells: the expression levels of Slc2a8/Glut8 and Slc2a12/Glut12 followed different temporal trajectories (see Fig 1).

SLC2A9/GLUT9 expression has been detected in human placenta [59], with two different splice forms [60]. Expression was found elevated in placentae of women with pre-gestational diabetes [27], and in kidney and liver in a diabetic mouse model [61]. In our study, however, there were no consistent responses of Slc2a9/Glut9 levels to the metabolic disease or to maternal diet. The incomplete prenatal lethality of homozygous Slc2a9/Glut9 knockout mouse embryos [62] would be consistent with a functional role of Slc2a9/Glut9 in placenta, although detailed investigations have not been performed.

Expression of Slc2a10/Glut10 and Slc2a13/Glut13 has been reported for human placenta [63, 64]. To our knowledge, our results are the first to provide temporal profiles for Slc2a10/Glut10 and Slc2a13/Glut13 expression in the mouse placenta.

### Modulation of fatty acid transporter gene expression in mouse placenta by diabetes and diet

The expression levels of fatty acid transporter genes in our study generally increased over the course of pregnancy, except for Slc27a5/Fatp5. The strongest increases were observed for Slc27a3/Fatp3 and Slc27a6/Fatp6, followed by CD36, Slc27a4/Fatp4, Slc27a1/Fatp1 and Slc27a2/Fatp2. Although their expression was influenced by the maternal metabolic condition at some time points, there was no consistent pattern of response. As found before for the glucose transporter gene family, the responses of fatty acid transporter expression to maternal diet also varied over time, with diet exerting stronger effects than diabetes.

Changes of fatty acid transporter gene expression in response to maternal nutritional status have also been reported for other systems: CD36 expression was increased compared to controls in a mouse model of IUGR [65], and also by high-fat diet feeding [66]. Slc27a6/Fatp6 was elevated in placenta from mice fed a high-fat-high-sugar diet [67]. In human pregnancies, CD36 levels were increased in IUGR-affected placentae, in which Slc27a6/Fatp6 expression was also higher than normal [68]. Both genes exhibited increased expression in human obese pregnancies [69] with CD36 elevated both at the mRNA and protein levels, while Slc27a4/Fatp4 was detected at lower levels [70]. In nutrient-restricted ewes, CD36 and Slc27a4/Fatp4 expression increased [71]; both genes also had elevated expression at the RNA level in obese ewes at midgestation [72]. On the other hand, in a rat model of diet-induced obesity that is associated with IUGR, mRNA and protein levels of CD36, Slc27a1/Fatp1 and Slc27a4/Fatp4 were reported as decreased relative to control [73]. It remains to be investigated to what extent altered transporter expression is linked to changes in fatty acid availability and metabolism.

Metabolism by ß-oxidation requires transport of fatty acids into the mitochondria, which for long-chain fatty acids is accomplished by the carnitine acyl transferases [74, 75]. According to our data, Cpt1a and Cpt2 are predominant in the mouse placenta, with an increase over pregnancy time for Cpt2. Responses to maternal diabetes were observed at some time points, but expression was influenced predominantly by diet. We observed higher Cpt1a expression in placentas from dams fed the breeder diet at E9.5, E10.5, and E15.5, and chow-feeding elevated Cpt1a expression at E12.5. In humans, high activity of Cpt2 was reported in chorionic villi and term placenta [76]. We observed an influence of diet on Cpt2 expression only at early stages of pregnancy. Although we currently cannot address whether the dynamic pattern of Cpt1a expression could reflect changes of the cell type composition, our combined results show that mitochondrial fatty acid transporter gene expression in the placenta responds to maternal diet, mostly at early stages of pregnancy.

### Relationship of nutrient transporter expression to placental and offspring weight in diabetic pregnancies

We previously showed that the placenta remains smaller in diabetic pregnancies in our experimental model, especially in dams fed the breeder diet [9], in which placenta weight on average was 15% lower at E15.5 and 10% lower at E18.5. Concomitantly, fetal size was smaller in diabetic dams, and the growth reduction was aggravated in breeder-diet-fed diabetic dams [10], where fetal length on average was 15% shorter at E15.5 and E18.5. A summary of observations for the E18.5 time point is presented in Table 2. We here sought to determine to what extent nutrient transport in the placenta is affected by metabolic status and diet, and whether the unfavorable fetal and placental outcomes could result from changes in the expression of nutrient transporters. Our results clearly show that nutrient transporter gene expression is sensitive to maternal diabetes and diet, particularly at earlier stages of pregnancy when the placenta is being established. But in late pregnancy, expression differences were observed for a few of the genes assayed here: Slc2a6/Glut6, Slc2a8/Glut8, CD36, Slc27a5/Fatp5, and Slc27a6/Fatp6. Expression levels of the latter three genes at E18.5 were moderately higher in placentae from non-diabetic dams, but there was no influence of diet at this time point. For the already low-levels of Slc2a6/Glut6 and Slc2a8/Glut8, the breeder diet was associated with approximately 2-fold reduced expression at E18.5, but there was no effect of maternal diabetes. Taken together with our finding that the majority of transporter genes do not display expression differences by metabolic status or diet at E18.5, our results do not reveal obvious correlations between transporter expression levels and placenta size (nor fetal size) shortly before birth. Our data for the E15.5 time point, including interaction analyses, are consistent with this notion. Thus, reduced placental and fetal growth in diabetic dams is unlikely to be the result of altered nutrient transporter expression during the second half of the pregnancy, at least as assayed at the steady-state mRNA level. While we cannot rule out the possibility that transporter protein activity might differ, these results raise caveats for using late pregnancy placental mRNA levels as proxy for pregnancy outcome.

**Table 2:** Phenotypes in the STZ-induced diabetic pregnancy FVB mouse model. Data for maternal weight gain, glucose levels, embryo length and placenta weight were taken from our respective prior publications (see References). There were no significant differences in litter sizes between experimental conditions.

Metabolic State	Controls		Diabetic		Reference
Diet	Chow	Breeder	Chow	Breeder	
Maternal weight gain E0.5 to E18.5 (in gr)	19.94 ± 2.69	18.61 ± 3.22	11.02 ± 3.56	12.95 ± 2.50	[9]
Maternal glucose level at E18.5 (in mg/dL)	118.0 ± 8.27	112.6 ± 22.08	484.8 ± 106.09^a^	581.6 ± 47.78^b^	[10]
Embryo size at E18.5 (length in mm)	30.85 ± 1.81	29.71 ± 3.78	26.99 ± 3.00	24.47 ± 3.89	[10]
Placenta weight at E18.5 (in mg)	61.65 ± 7.15	60.82 ± 8.24	64.58 ± 5.21	53.3 ± 7.95	[9]

^a^) 1 out of 10 dams had a glucose level of equal or greater than 600 mg/dL

^b^) 10 of 12 dams had a glucose level of equal or greater than 600 mg/dL

### Metabolic and diet influences on placenta formation are critical to developmental programming of adult susceptibility to disease

Importantly, our data show that at earlier stages of pregnancy, nutrient transporter gene expression during formation of the placenta is indeed modulated by maternal diabetes and maternal diet. These results augment our earlier findings of strong influences and interactions of metabolic status and diet on other placental genes as early as E10.5 [9]. We here report gene expression profiling at even earlier time points during placenta formation (E8.5 and E9.5), which also respond strongly to differences in maternal diet and diabetes. These changes of gene expression before and at mid-gestation temporally precede the reduced placenta growth in breeder-fed diabetic dams, which is apparent by E12.5. Thus, our results highlight the importance of expression changes in the first half of pregnancy for placental and fetal outcomes near term. Yet, it remains to be investigated how altered nutrient transporter expression affects rates of transport and availability of specific nutrients for growth of the placenta and conceptus. Interactions between diet and metabolic conditions on gene expression levels at early pregnancy time points (E9.5 and E10.5) suggest several genes in the glucose transporter family that could be involved in reduced placenta growth in diabetic pregnancies, i.e. Slc2a1/Glut1, Slc2a5/Glut5, Slc2a6/Glut6, Slc2a8/Glut8, Slc2a9/Glut9, Slc2a10/Glut10, Scl2a12, and Slc2a13; the previously unrecognized effects of maternal diabetes on the expression of fatty acid transporter genes Slc27a1-a5/Fatp1-5, and mitochondrial fatty acid transporter genes Cpt1a and 1b in particular, deserve further investigation. Thus, gene expression changes during placenta formation in the first half of pregnancy predicate the capacity for growth of the placenta during the second half of pregnancy in the mouse. These findings imply that metabolic programming effects by maternal diabetes and diet, insofar as they are related to placenta size, have their origin prior to and during formation of the placenta. Most importantly, this places the critical time window for programming of adult disease susceptibility earlier than previously postulated [7, 77, 78].

In summary, we have shown that even under conditions of maternal metabolic disease, placental nutrient transporter gene expression is predominantly responsive to and influenced by maternal diet, as evidenced by our comparison of two widely used commercially available mouse diets in a mouse model of diabetic pregnancy. Furthermore, this study highlights that -at the molecular level- the process of placenta formation is exquisitely sensitive even to moderate changes in maternal diet composition, particularly in the context of maternal metabolic disease.
